# A review of changes in COVID-19 burden in the COVID-19 treatment centres in Yaoundé (Cameroon): a call for cautious optimism

**DOI:** 10.11604/pamj.supp.2020.35.2.24982

**Published:** 2020-07-14

**Authors:** Eugene Sobngwi, Charlotte Omgba-Moussi, Charles Kouanfack, Noel Vogue, Alain Patrick Tchatchoua, Pierre Ongolo Zogo

**Affiliations:** 1 University of Yaoundé 1, Yaoundé, Cameroon,; 2 Yaoundé Central Hospital, Cameroon,; 3 Incident Management Team, Centre Region, Ministry of Public Health, Cameroon,; 4 Scientific advisory board of public health emergencies, Cameroon,; 5 University of Dschang, Cameroon

**Keywords:** COVID-19, Africa, early treatment, strategy, bimodal, mitigation curve

## To the Editors of the Pan African Medical Journal

Since the first case of pneumonia of unknown cause originating from China was reported to the WHO in December 2019, the COVID-19 pandemic has claimed more than 11,874,226 cases and 545,481 deaths worldwide [1]. In affected countries, the COVID-19 epidemic typically evolves rapidly, within weeks, reaches a peak and then follows a trend reflecting effectiveness of the response measures. The shape of the epidemic curves reflects the efficacy (or lack of it) of mitigation strategies implemented at country or local levels [2]. Cameroon, a country in Central Africa, reported its first RT-PCR confirmed case of COVID-19 on 5th March 2020 imported from France. For the four weeks following the notification of the first case, the outbreak was predominantly driven by imported cases. By the 8th week (early May), the country entered the community transmission phase affecting mostly the 10 administrative regions of the country with demonstrated community. As of 30 June 2020, all the ten regions of the country were affected. The early response strategy was based on a predominantly centralized incident management structure. On March 17, the government adopted 13 measures restricting population mobility and public gatherings within the country and across the borders with effect on April 9th; wearing of face masks in public places was also made compulsory. On 30th April however, some of the restrictions were loosen, including public gatherings in pubs and bars.

**Figure 1:**
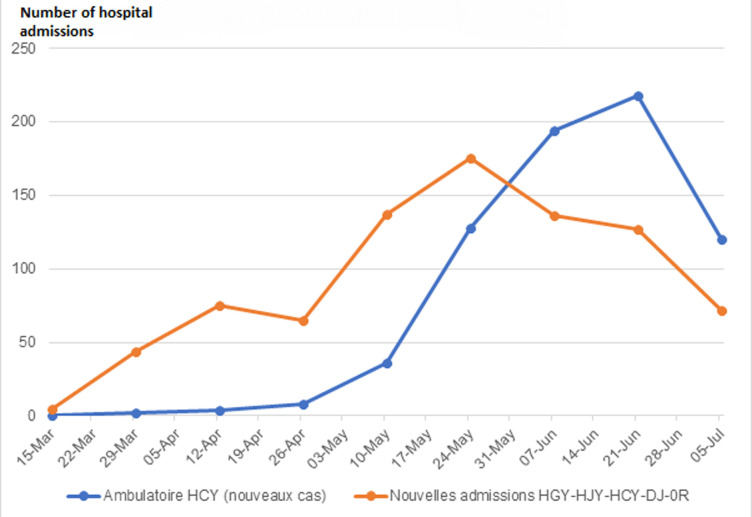
frequency of new hospital admissions and ambulatory follow up of COVID-19 cases in Yaoundé, Centre Region, Cameroon

Following the reopening of pubs and bars, which was perceived by most as the end of the epidemic, there was a rapid increase in reported COVID-19 cases and deaths; from week 8 to week 12 within the outbreak (Figure 1), the number of COVID-19 cases increased from 2000 to over 8000, and from 60 to 200 respectively, during the month of May 2020 (data not shown) [3]. This increase in cases and deaths due to the relaxation of social distancing measure and the reopening of bars and pubs is inferred and not established. This worsening of the pandemic in the country called for a review of the response strategy with emphasis on decentralisation of all activities especially the creation of regional incident management systems and laboratory facilities for RT-PCR and rapid antigen testing to bring the services closer to the communities. Extensive contact tracing, testing, and early treatment served as the backbone of the revised strategy. The decentralized strategy, in addition to expanded testing capacities also included treatment of all positive cases at primary care and community level. While there is no globally approved treatment for asymptomatic and mildly symptomatic cases of COVID-19, the Cameroon Public Health Emergencies Scientific Council revised the national clinical case management guidelines to include low dose and short-term hydroxychloroquine, azithromycin-based treatment plus zinc for all COVID-19 cases including asymptomatic and mild symptomatic. These revised guidelines were adopted on May 13th, 2020 and circulated to all health facilities of the country.

This paper describes the changes in COVID-19 hospital admission in Yaoundé from 15 March to 5 July 2020, with data from five dedicated COVID-19 treatment centres (Annex 1) and COVID-19 surveillance database. Figure 1 shows the change in COVID-19 hospital visits and admissions in the five COVID-19 treatment centres of the capital City Yaoundé from 15 March to 5 July 2020. The graph shows a marked increase in new admissions of severe cases of COVID-19 from 15 March to 27 May followed by a rapid decrease that has been sustained until the latest data available prior to the finalization of this reports (5 July). This evolution suggests that the region reached a peak in admissions around the third week of May (10th week of the epidemic). In fact, by May 24th, the bed occupancy in specialised COVID care units in tertiary hospitals reached 122%, prompting the country to open large capacity specialised centres in regional capitals. Bed occupancy in the five specialised COVID care units has since decreased to reach its lowest level (24%) as of July 5th. Overall, hospital admissions for COVID-19 reached a peak around the 10th week (Week of 24 May) while the number of ambulatory visits related to COVID-19 reached a maximum around the 13th - 14th week (16-22 June).

**Figure 2:**
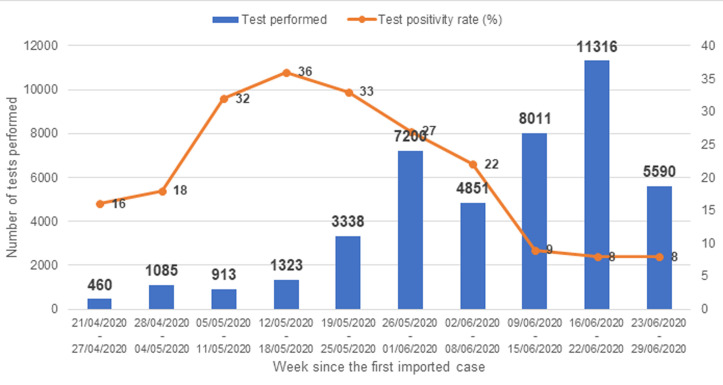
number of COVID-19 tests performed and test positivity rates in the Centre Region of Cameroon over the period of interest Variations of confirmatory tests positivity rate

A review of testing data following the country expansion of COVID-19 testing capacity showed a rapid increase in confirmed (positive) cases, this increase was however not associated with the expected increase in the severe forms of the disease, on the contrary, a reduction in the severe forms requiring specialized hospital care was noted. The expansion of testing also resulted in a marked reduction of positivity rate (i.e. the ratio of people tested positive over the total number of people tested) over time. Culminating around 29% on average over the first 12 weeks on antigenic testing (RT-PCR or rapid antigenic testing) with over 12000 confirmed cases, the positivity rate decreased to 8% on average over the 14th and 15th week (Figure 2). It should be noted that early in the pandemic, testing was limited primarily to suspected cases (close contacts of confirmed cases); as testing became more available and less selective, more people were tested; hence the reduction in the observed positivity rate.

**Annex 1:**
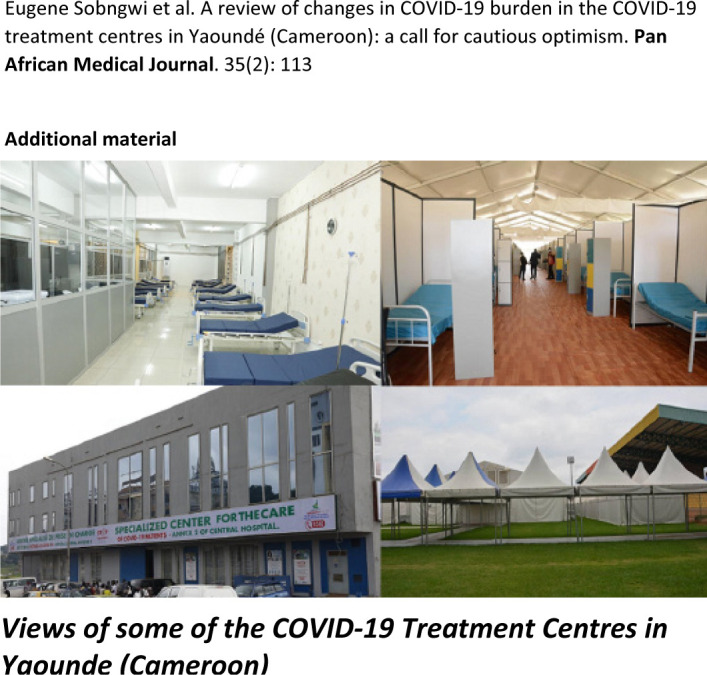
photos of the COVID-19 treatment centers

This COVID-19 trend in hospital admissions and ambulatory care for CIVID-19 in the capital city of Cameroon at this stage of the pandemic is peculiar and unexpected considering the non-optimal adherence by population to protective measures and the increased relaxation of physical distancing restrictions. Possible explanations to this phenomenon include a change in health seeking behavior from COVID-19 patients who prefer to seek health care outside of official treatment centers in Yaoundé, a shift of the outbreak to younger age groups less susceptible to the severe forms of the disease, or a beneficial effect of early treatment of positive cases. More research is needed to assess the current medical burden of COVID-19 in Yaoundé. It will be premature however to anticipate an end to the outbreak in Yaoundé, vigilance should be maintained. All the pillars of the current interventions and continue preparedness for possible surge in COVID-19 cases should be sustained; with emphasis on strengthening district health system to ensure they are responsive and deliver people-centered services, tailored to contexts and territories, and fit for diverse stages of the pandemic.

**Ethics committee approval:** the study was approved by the National Ethics Committee, Cameroon under Number 2020/05/1505/L/CNERSH/SP.

## References

[1] World Health Organization WHO Coronavirus Disease (COVID-19) Dashboard.

[2] Vicentini C, Bordino V, Gardois P, Zotti CM (2020). Early assessment of the impact of mitigation measures on the COVID-19 outbreak in Italy. Public Health.

[3] UNICEF Cameroon: COVID-19 Situation Report #12.

